# Optimization of Normal Human Bronchial Epithelial (NHBE) Cell 3D Cultures for *in vitro* Lung Model Studies

**DOI:** 10.1038/s41598-018-36735-z

**Published:** 2019-01-24

**Authors:** Rachael E. Rayner, Patrudu Makena, Gaddamanugu L. Prasad, Estelle Cormet-Boyaka

**Affiliations:** 1 The Ohio State University, Department of Veterinary Biosciences, Columbus, OH 43210 USA; 2 RAI Services Company, Winston-Salem, NC 27101 USA

## Abstract

Robust *in vitro* lung models are required for risk assessment to measure key events leading to respiratory diseases. Primary normal human bronchial epithelial cells (NHBE) represent a good lung model but obtaining well-differentiated 3D cultures can be challenging. Here, we evaluated the ability to expand primary NHBE cells in different culture conditions while maintaining their 3D culture characteristics such as ciliated and goblet cells, and ion channel function. Differentiated cultures were optimally obtained with PneumaCult-Ex Plus (expansion medium)/PneumaCult-ALI (differentiation medium). Primary cells passaged up to four times maintained airway epithelial characteristics as evidenced by ciliated pseudostratified columnar epithelium with goblet cells, trans-epithelial electrical resistance (TEER) (>400 Ohms.cm^2^), and cystic fibrosis transmembrane conductance regulator-mediated short-circuit currents (>3 µA/cm^2^). No change in ciliary beat frequency (CBF) or airway surface liquid (ASL) meniscus length was observed up to passage six. For the first time, this study demonstrates that CFTR ion channel function and normal epithelial phenotypic characteristics are maintained in passaged primary NHBE cells. Furthermore, this study highlights the criticality of evaluating expansion and differentiation conditions for achieving optimal phenotypic and functional endpoints (CBF, ASL, ion channel function, presence of differentiated cells, TEER) when developing *in vitro* lung models.

## Introduction

Mucociliary clearance in the human lungs is the first line of defense to continuously remove harmful aerosols, pathogens and toxins. Healthy airways consist of a pseudostratified epithelium containing four primary cell types: basal stem cells which anchor the basement membrane, ciliated cells (50–70%) which use motile force to remove trapped particles and pathogens in the mucus, mucus (MUC5AC and MUC5B) producing goblet cells (~25%), and Club cells (~11%) expressing club cell-specific 10 kDa (CC10) secretory protein with anti-inflammatory and immune-modulating properties^1,2^. A crucial balance of salt and water must be secreted into the airway surface liquid (ASL) to maintain mucus clearance. Disruption to this first line of defense can be caused by air pollution, genetic mutations, cigarette smoking, and pathogenic infections associated with diseases such as chronic obstructive pulmonary disease (COPD) and cystic fibrosis (CF)^3–6^. According to the World Health Organization (WHO), approximately 3 million deaths were due to COPD in 2015 (5% of all deaths globally in that year), and most of these deaths (90%) occur in low or middle-income countries. Therefore, development and validation of *in vitro* models to assess biological responses against disruption of the first line of defense in upper airways is crucial.

3D primary human airway epithelial cultures are increasingly used as an *in vitro* model for biological assessments to investigate function and mechanisms as well as to minimize the use of *in vivo* animal models. Airway-derived cell-lines form monolayers (such as 16HBE and Calu-3) but do not consist of multiple differentiated cells, including ciliated and goblet cells, and may be more sensitive to treatment/disruptions. They are also often cancer-derived such as A549 and Calu-3 cells. A recent review by Fang and Eglen^7^ clearly outlines the limitations of 2D cellular cultures, and highlights the current 3D models. However, the use of primary cells for culturing 3D epithelial layers also presents its own limitations including their availability and price, rate of cellular expansion, and the number of time cells can divide. In addition, obtaining well-differentiated cultures can be challenging. Hence, most of the published studies use cells passage one or two even though some commercial primary cells are guaranteed to reach 10–15 population doublings (about 4 passages) before expansion rates dramatically decline, and differentiation potentially fails. It is possible to immortalize primary human adult cells, such as with exogenous human telomerase reverse transcriptase (hTERT), viral oncoproteins (HPV-16 E6 and E7, or SV40 T-antigen), defective SV40 virus genome and additional cellular genes such as *cdk4*^8–16^. However, resulting cells can have disrupted differentiation or lack crucial biomarkers typical of an *in vivo* airway epithelium, for example BEAS-2B cells may not express MUC5AC, the goblet cell secreted protein that plays a crucial role in mucociliary clearance^17^.

Maintenance of normal phenotypic characterization and function of airway epithelial cells when passaging primary cells is essential. Key strategies to ascertain epithelial polarization include: (a) measurement of trans-epithelial electrical resistance (TEER) across the epithelial layer, (b) expression and localization of tight junction proteins by immunofluorescence (e.g. ZO-1), or (c) assessment of the diffusion of small tracer molecules^18^. Due to the key function of the ion channel CFTR in airway fluid homeostasis and therefore, mucociliary clearance, assessment of its function will also further enhance our knowledge of the primary 3D airway epithelium *in vitro* model^19–21^. To date, it is unknown whether passaged primary 3D airway epithelial cultures retain functioning CFTR channels. Other important features of mucociliary clearance include ciliary beat frequency (CBF), airway surface liquid (ASL) and presence of mucin-secreting goblet cells. The crucial steps that can determine the success of growing primary human cells into fully differentiated pseudostratified epithelium include the expansion and differentiation media, which contain numerous chemical and biological reagents. A number of commercial vendors can provide such media. Published research has furthermore attempted to extend cell life and differentiation potential by investigating the addition of growth-inactivated keratinocytes as fibroblast feeder cells and a Rho-associated kinase (ROCK) inhibitor, Y-27632^22–27^.

In an attempt to understand how the culture conditions affect cell growth, epithelial phenotype and function, we investigated the effect of expansion media and passage numbers on primary NHBE cells cultured at air-liquid interface (ALI). Several epithelial features were assessed, including bronchial epithelial phenotype, culture characteristics, cell integrity, differentiation, and function of ion channels.

## Results

### Optimal expansion of NHBE cells was achieved with PneumaCult-Ex Plus (M3) or BEGM (M1) medium with ROCK inhibitor

**Figure 1:**
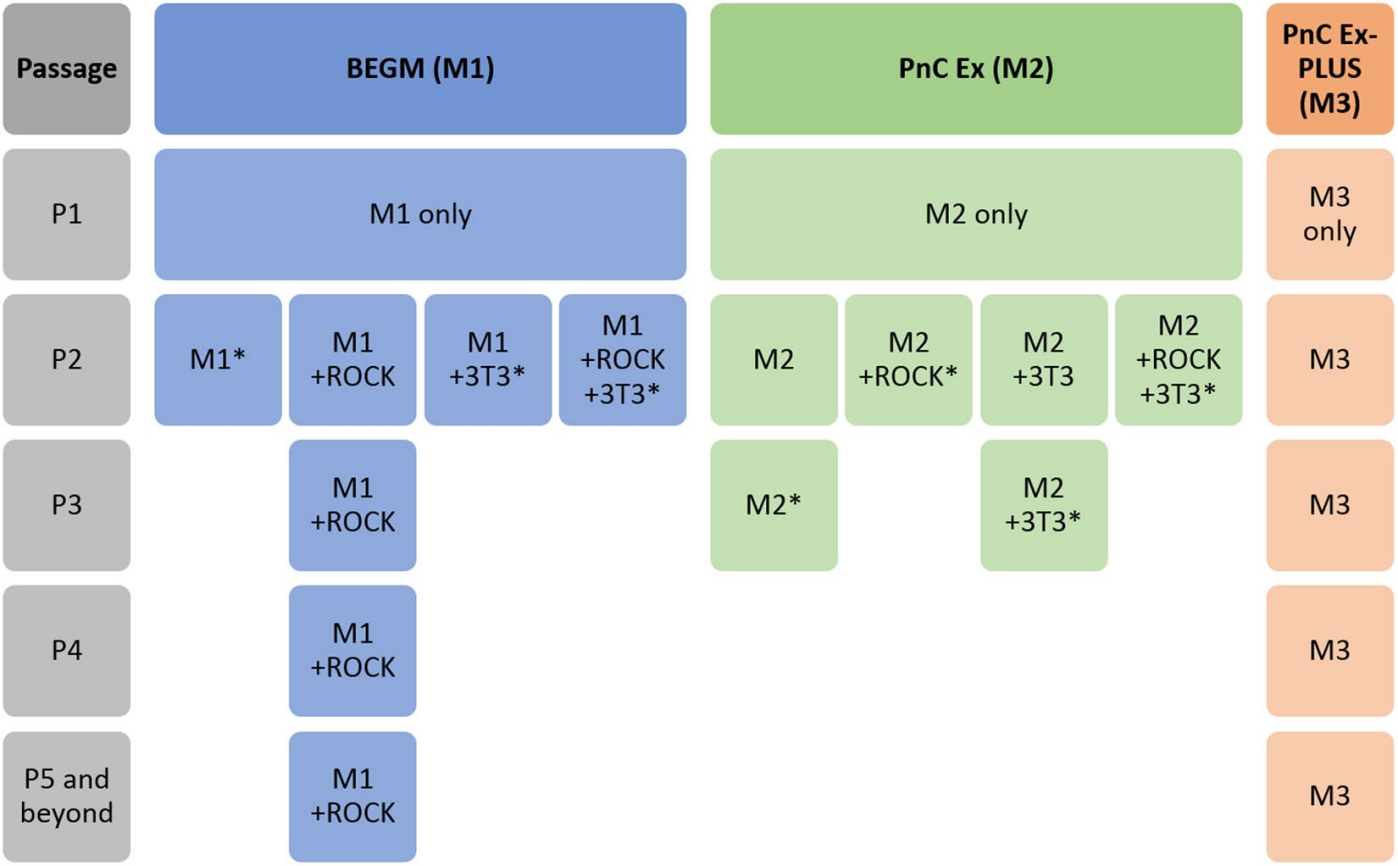
Expansion of primary NHBE cells in three different expansion media prior to ALI. NHBE cells were cultured in three different expansion media from passage one (P1), BEGM medium (M1), PneumaCult-Ex medium (M2) or PneumaCult-Ex Plus medium (M3). Rho kinase inhibitor (ROCK) and/or 3T3 Swiss albino feeder cells (3T3) were added to growth media conditions from passage two (P2) in only the BEGM (M1) or PnC-Ex media (M2).*Cells were not passaged further in these conditions if they took longer than 7 days to reach ~75% confluency.

Primary NHBE cells were originally expanded in three different growth media at passage one – BEGM (M1), PneumaCult-Ex (M2) or PneumaCult-Ex Plus (M3) (Fig. 1 and Fig. S1). Cells readily proliferated; however, it was noticeable that cells grown in M3 medium appeared to form typical cobble stone-shaped clustered ‘colonies’, contributing to a higher density of cells when reaching 70–80% confluency (Fig. S2A,B). From passage two, NHBE cells were either expanded in media alone, or with the addition of ROCK inhibitor or 3T3 Swiss albino feeder cells (3T3). ROCK inhibitor and 3T3 feeder cells were only tested with M1 and M2 media. We chose to only test the ability of M3 medium to proliferate cells by itself since clustered colonies were observed in absence of 3T3 cells or ROCK inhibitor. NHBE cells grown in M2 media, or variations thereof, were only grown until passage four (P4) since it was already clear that M1 + ROCK and M3 were more proficient for cell growth and expansion. Beyond passage five (P5), NHBE cells were only grown in either M1 + ROCK or M3 media (Fig. 1). Various differences in proliferation emerged depending on the growth medium and the additional factors, for example NHBE cells proliferating in M1 medium appeared to excel with the addition of ROCK but not 3T3 feeder cells (Fig. S2A,B). NHBE cells were expanded up to eight passages in M3 medium, whereas NHBE cells continued up to passage twelve in M1 + ROCK medium (Fig. S3A,B). However, the ability for these passaged NHBE cells to form 3D epithelial cultures needed to be determined.

### Optimal differentiation of NHBE cells was achieved from PneumaCult-Ex Plus (M3) grown cells

**Figure 2:**
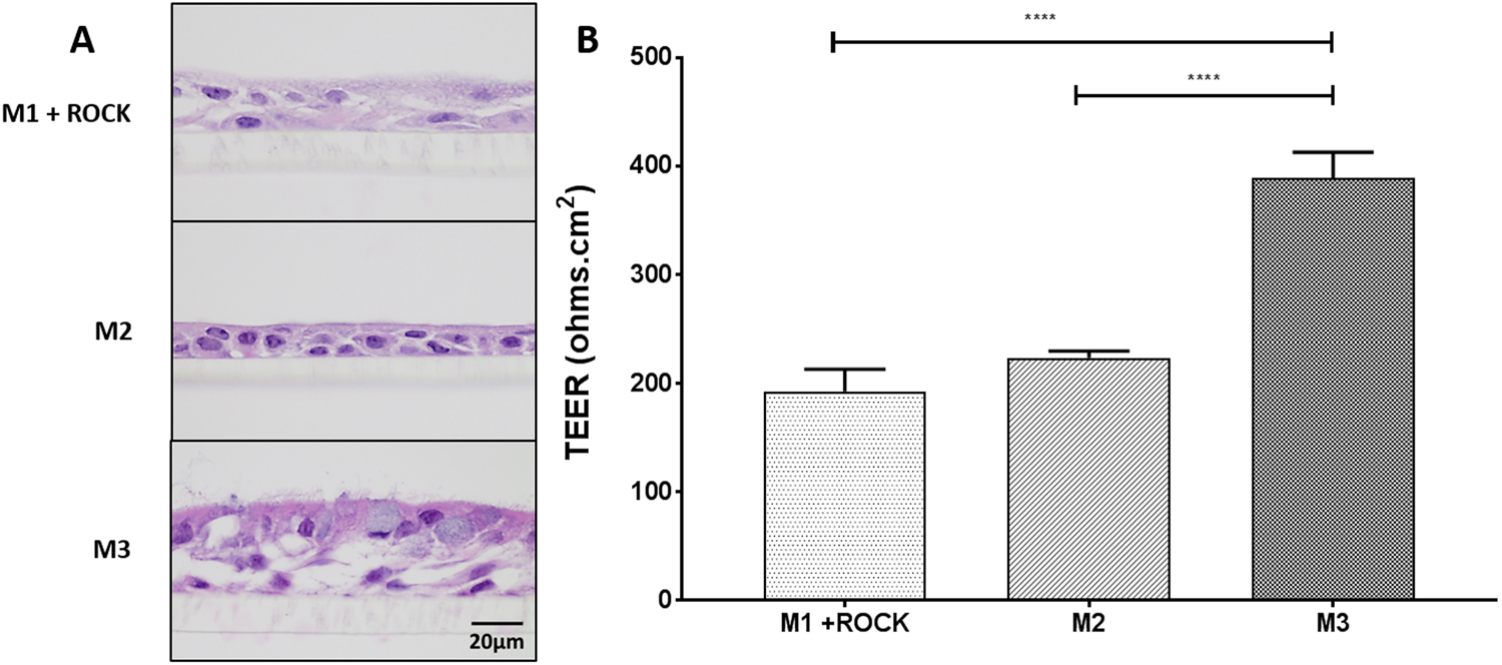
Differentiation of primary NHBE cells at ALI with three different culture conditions. NHBE cells at passage two were cultured at ALI for 4 weeks in different growth media. The resulting 3D cultures were stained with Hematoxylin and Eosin (H&E). (**A**) H&E staining of NHBE layers grown from cells cultured in M1 + ROCK (BEGM + ROCK), M2 (PnC Ex) or M3 (PnC-Ex-PLUS). (**B**) TEER measurements of epithelial layers grown in ALI from cells expanded in M1 + ROCK, M2 or M3 media. ****p < 0.0001.

**Figure 3:**
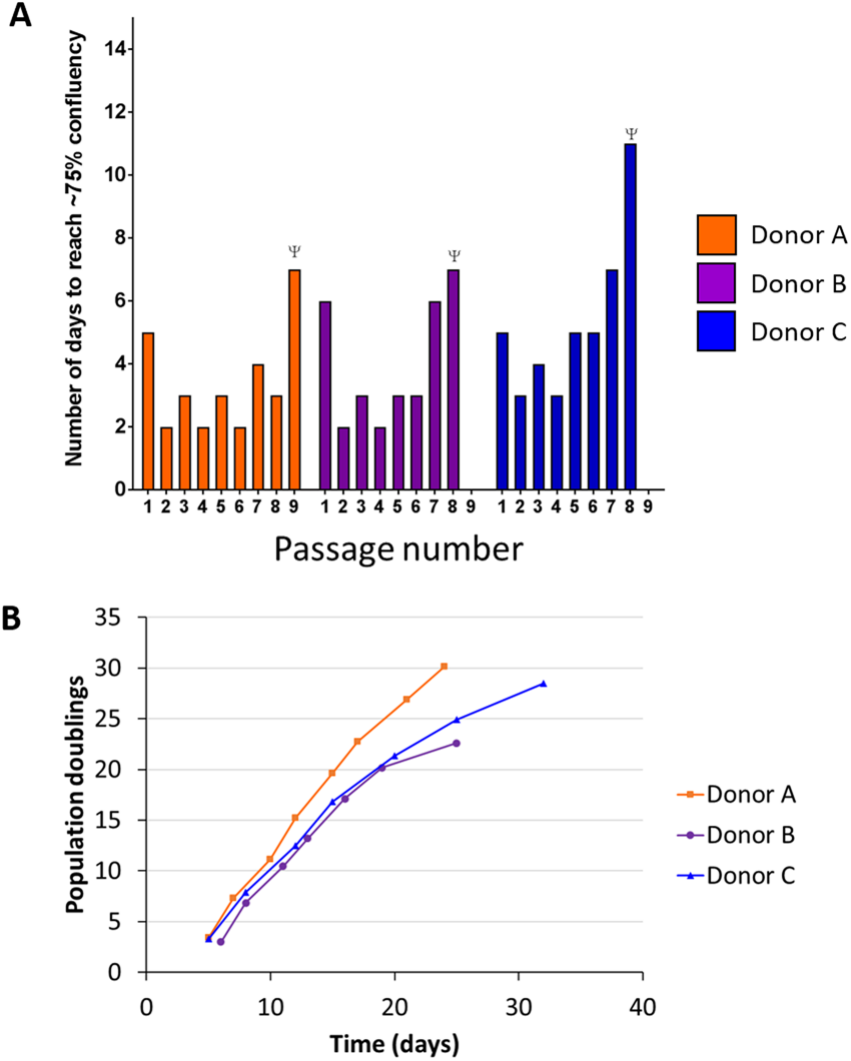
Expansion of primary NHBE cells in M3 (PnCEx-PLUS) expansion medium. Expansion of NHBE cells from three different donors was achieved using M3 expansion medium. (**A**) Primary NHBE cells could be expanded up to eight passages before growth declined. (**B**) Population doublings were fairly consistent up to the final passage number. Primary NHBE were expanded 8 times within 30 days for most donors. ^Ψ^Cells at this passage failed to reach 70–80% confluency within 7 days.

Despite successful growth of NHBE cells in M1 + ROCK, M2 or M3 media, the ability for the cells to differentiate into 3D epithelial cultures was best achieved with growth-expansion M3 medium prior to culturing at ALI in PneumaCult-ALI (ALI) medium for 4 weeks (Fig. 2). Histology of the 3D cultures demonstrated that M1 + ROCK and M2 media only resulted in a 2–3 multi-layered epithelium with sparse differentiated cells within 4 weeks (Fig. 2A**)**. Cells grown in M2 medium eventually formed a thicker epithelium with differentiated cells when time at ALI was extended to 8 weeks (Fig. S4). However, cells grown from M3 medium differentiated into goblet and ciliated cells, forming a thick 4–6 multi-layered epithelium within 4 weeks at ALI. Trans-epithelial electrical resistance (TEER), which is a strong indicator of epithelium integrity, indicated the polarization of cells grown from M3 but not M1 + ROCK nor M2 media (Fig. 2B). M3 medium was therefore used to expand NHBE cells from three different donors (Fig. 3A,B). From passage two to six, cells reached about 75% confluence in less than 6 days per passage. After passage six, the number of days to reach confluence increased, indicative of a decline in cell growth (Fig. 3A). Overall, primary NHBE cells could be cultured up to 8 times within 30 days for most donors (Fig. 3B).

We next evaluated the effect of cell density on 3D cultures prior to ALI (M3 growth medium used for seeding cells). Optimal seeding density of cells onto transwells was determined at 50,000 cells/well in M3 growth medium (Fig. S5B). At higher seeding densities (100,000 cells/well), very thick epithelial layers were formed (Fig. S5A), possibly due to overcrowding of cells in transwell. Lower seeding densities (25,000 cells/well) could still form epithelial layers, although epithelial multilayer was only 2–3 cells thick (Fig. S5C).

### Passaged primary NHBE cells grow into pseudostratified airway epithelial layers

**Figure 4:**
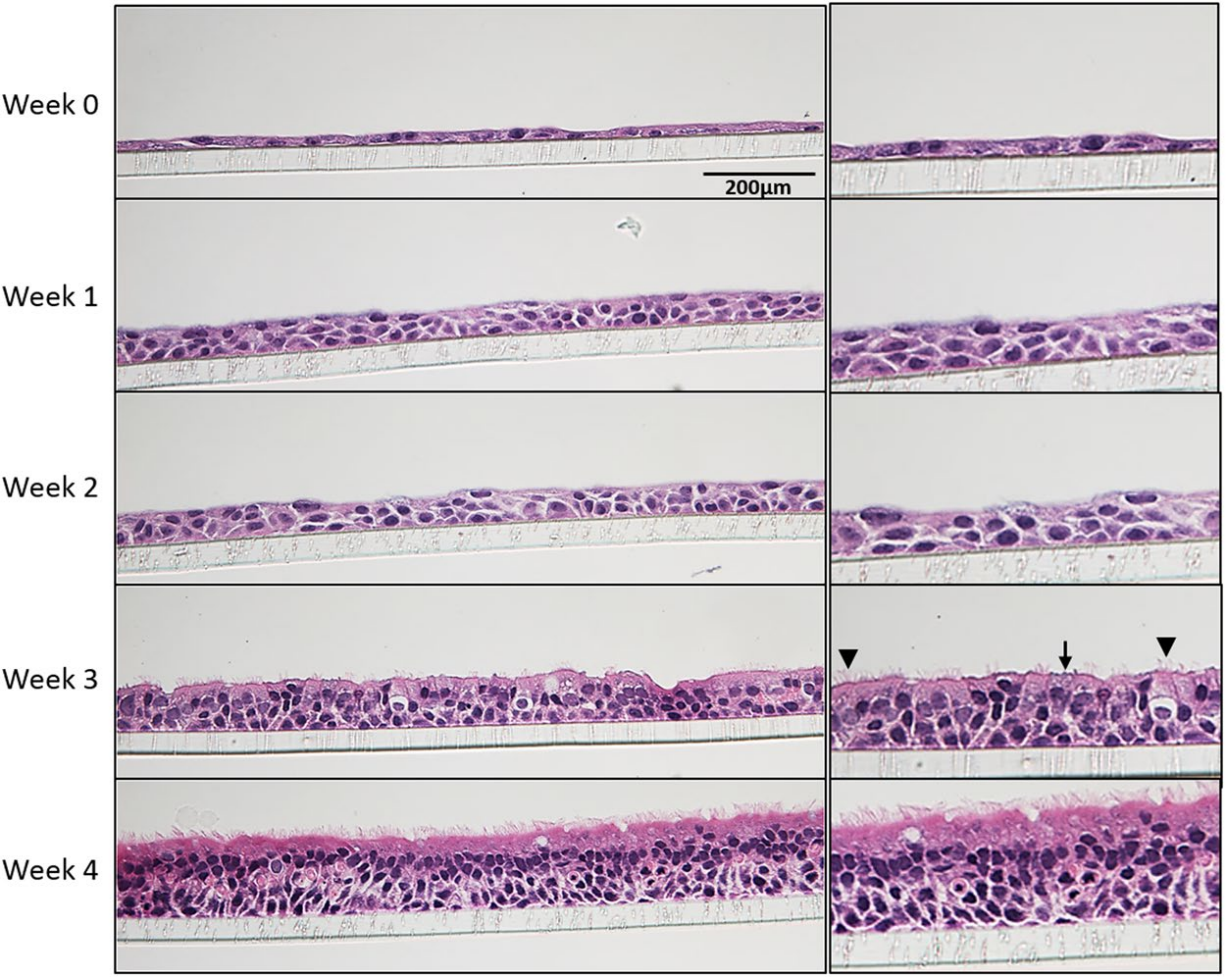
Development of 3D cultures over 4 weeks at ALI. NHBE cells (Donor C, passage 1 taken directly from cryopreservation) were grown for up to 4 weeks in M3 medium. 3D cultures were collected each week after being air-lifted and subjected to H&E staining. Both goblet (arrow) and ciliated cells (arrowhead) can be detected at 3 weeks. Objective X400.

A fully differentiated pseudostratified epithelial layer was determined to be achieved by 4 weeks at ALI (Fig. 4). Beginning with a confluent monolayer of NHBE cells (week 0) submerged in M3 medium, the cells were airlifted (apical medium removed and basal medium switched to PneumaCult-ALI). Within one week, the epithelial layer was at least 2–3 cells thick, but differentiated goblet or ciliated cells were not observed by histology. Ciliated and goblet cells were visualized by 3 weeks ALI, and a thick epithelium was observed with columnar ciliated cells and goblet cells after 4 weeks ALI.

**Figure 5:**
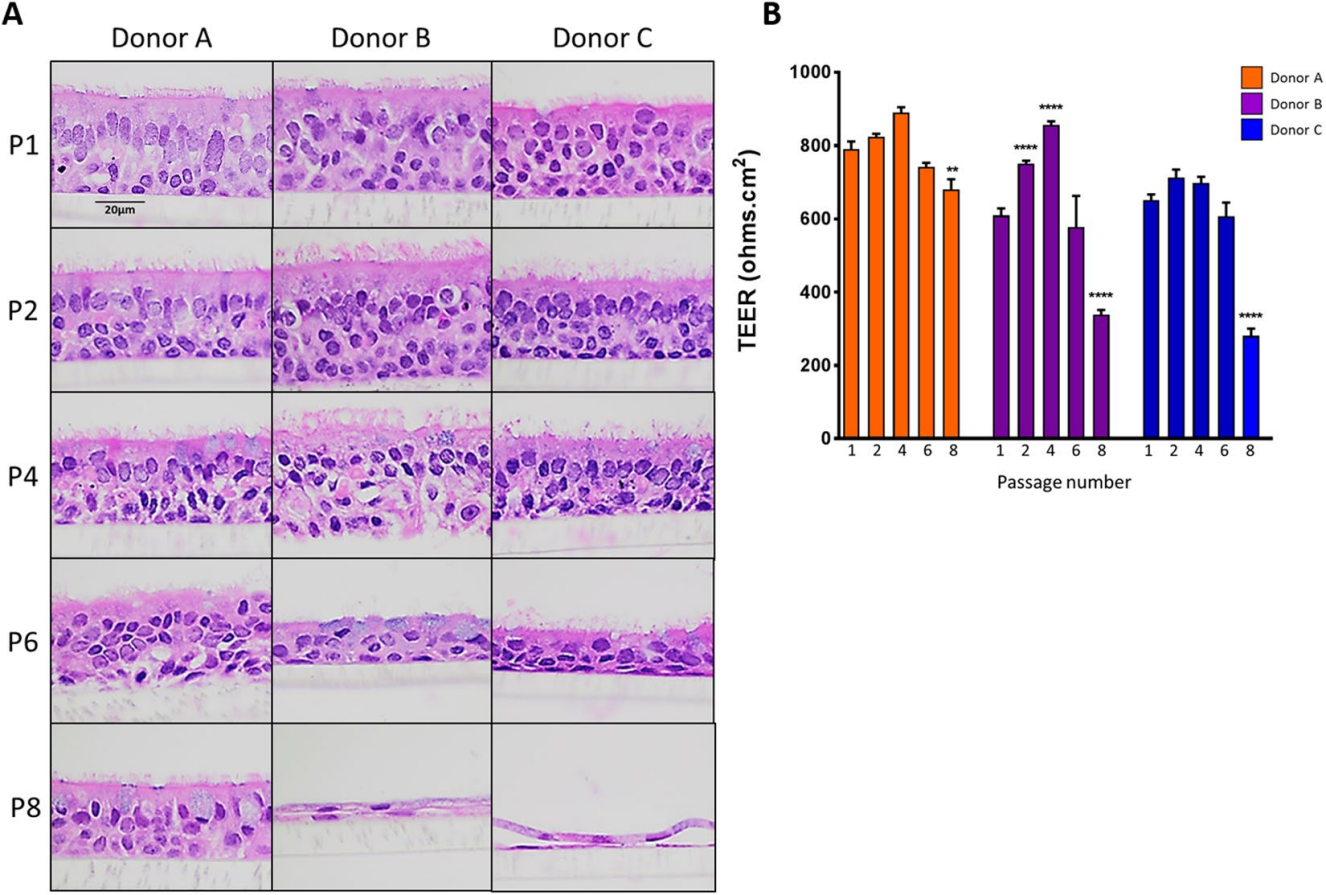
Differentiation of primary NHBE cells from passaged cells grown in M3 (PnC-Ex-PLUS) expansion medium. NHBE cells expanded in M3 expansion medium to passage 1, 2, 4, 6 and 8 were cultured at ALI for 4 weeks in ALI differentiation media. The resulting 3D cultures were stained with H&E. (**A**) H&E staining of passaged NHBE cells (4 weeks ALI; X600 objective). (**B**) Trans-epithelial electrical resistance (TEER) of 3D cultured NHBE cells, 4 weeks ALI. Significance compared to first passage. **p < 0.01, ****p < 0.0001.

Next, we assessed the ability of passaged NHBE cells expanded in M3 medium (passages 1, 2, 4, 6 and 8) to form 3D cultures (Fig. 5A). Pseudostratified, fully differentiated epithelial layers were formed within 4 weeks from NHBE cells passaged up to 4 times from three different donors. Beyond passage four, the epithelial layers began to lose their columnar ciliated cell structure in most of the donors despite presence of cilia and goblet cells. 3D epithelial cultures could still be grown from passage eight NHBE cells from one donor (Donor A) but not the other 2 donors. TEER was measured to determine the integrity of the epithelial layers (Fig. 5B). All of the differentiated 3D cultures had a TEER ≥ 400 Ω.cm^2^ up to passage six. However, significant declines in TEER were observed for 3D cultures grown from these NHBE cells expanded beyond passage six. Interestingly, a wide range of TEER values have been reported for primary bronchial cells (400–4000 Ω.cm^2^), even as low as 150 Ω.cm^2 ^^18^.

**Figure 6:**
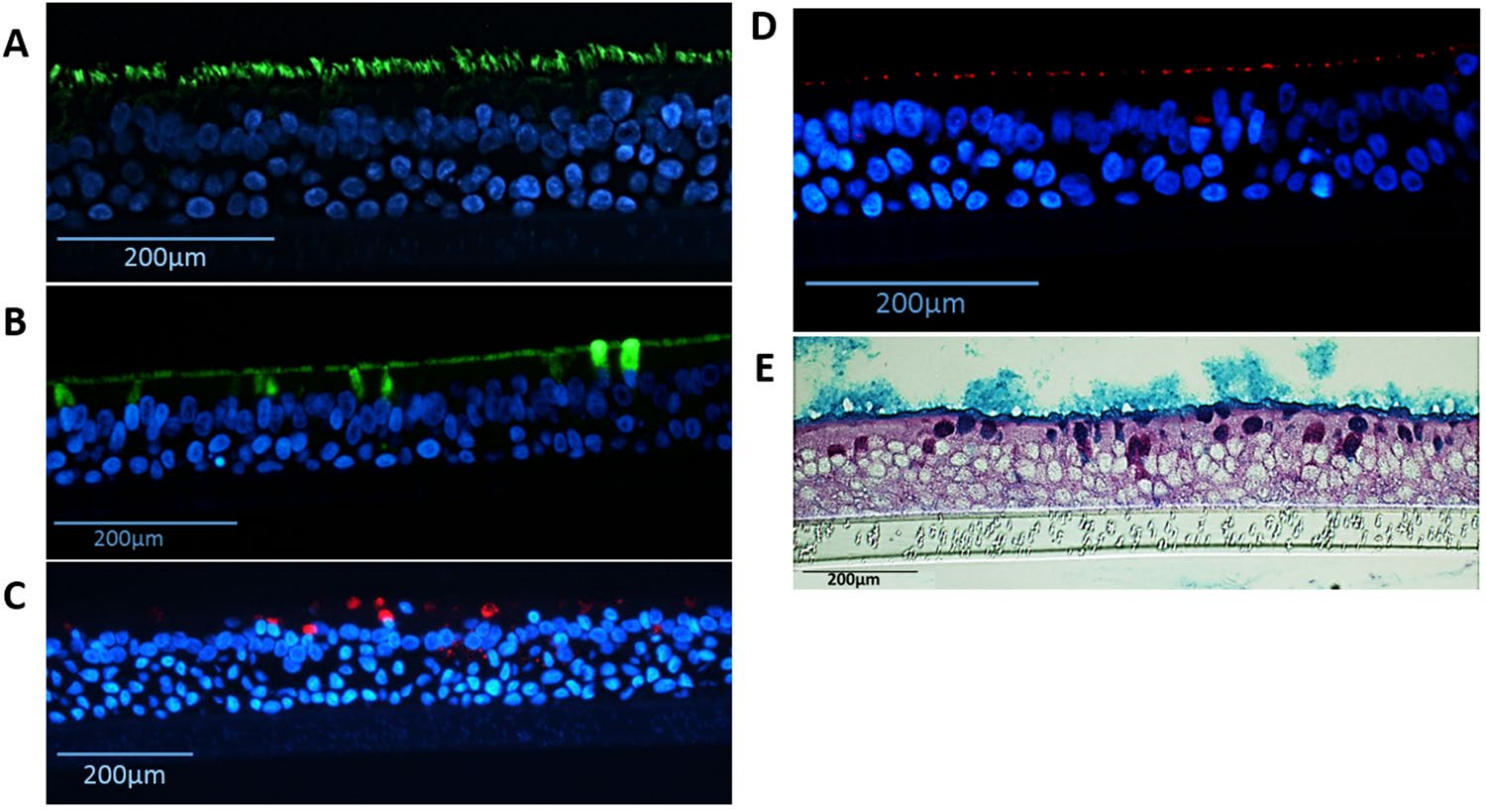
Detection of differentiated cells in NHBE pseudostratified epithelial layer generated from passage 1 cultures. Immunofluorescence for (**A**) Ciliated cells (α-tubulin, green), (**B**) goblet cells (MUC5B, green), (**C**) goblet cells (MUC5AC, red), (**D**) ZO-1 tight junctions (red), nuclear stain (DAPI - blue); and staining for  (**E**) periodic acid-Schiff/alcian blue for mucus producing goblet cells.

The presence of ciliated and goblet cells with tight junctions were identified using immunofluorescence and periodic acid-Schiff/alcian blue (PAS/AB) staining (Fig. 6). Ciliated cells were identified from the staining of α-tubulin on the apical surface (Fig. 6A), whereas both MUC5AC and MUC5B were identified for the presence of goblet cells (Fig. 6B,C). 15–26% of the cells were labeled with the marker of goblet cells MUC5B (Fig. S6). Tight junction protein ZO-1 was located near the apical surface, connecting the columnar cells together (Fig. 6D and Fig. S6). Figure 6E shows that mucins were present in goblet cells and covered the surface of the epithelium using PAS/AB staining.

### Ion channel function of CFTR but not ENaC is retained in primary NHBE 3D cultures from passaged cells

**Figure 7:**
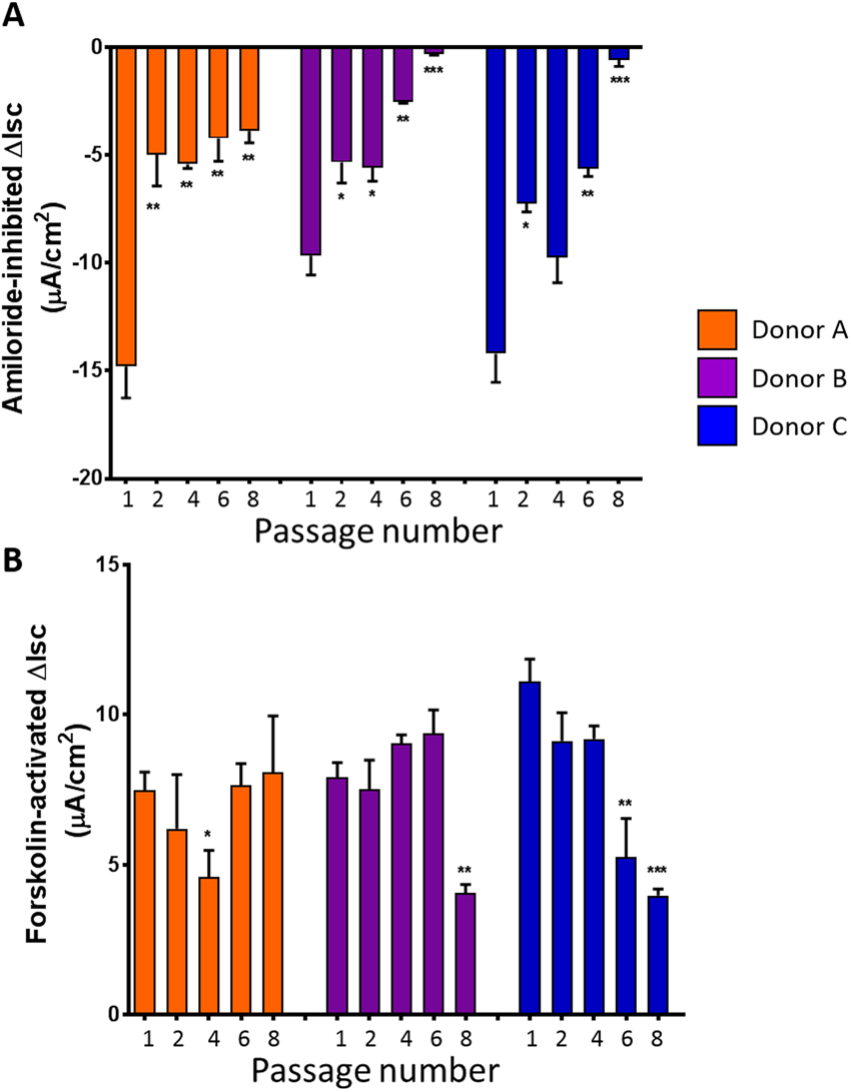
Effect of passage number on ENaC and CFTR channel function. Ion channel function of ENaC and CFTR were measured in NHBE 3D cultures upon reaching 4 weeks at ALI. (**A**) Amiloride inhibited currents were determined as described in the Methods section. (**B**) Forskolin-activated CFTR channels were measured as described in the Methods section. Data collated from 3 different donors, n = 3 for each donor. Significance compared to Passage 1, *p < 0.05, **p < 0.01, ***p > 0.001.

One crucial aspect when evaluating the airway models is the presence of functional ion channels, such as the epithelial sodium channel (ENaC) and the cystic fibrosis transmembrane conductance regulator (CFTR) channel. Surprisingly, ENaC function significantly declined in passaged cells beyond passage one in all donors (Fig. 7A). On the other hand, no significant decline in CFTR function was observed in epithelial layers grown from passaged NHBE cells up to passage six in most donors (Fig. 7B).

### Airway surface liquid (ASL) and ciliary beat frequency (CBF) is maintained in primary NHBE 3D cultures grown from passaged cells

**Figure 8:**
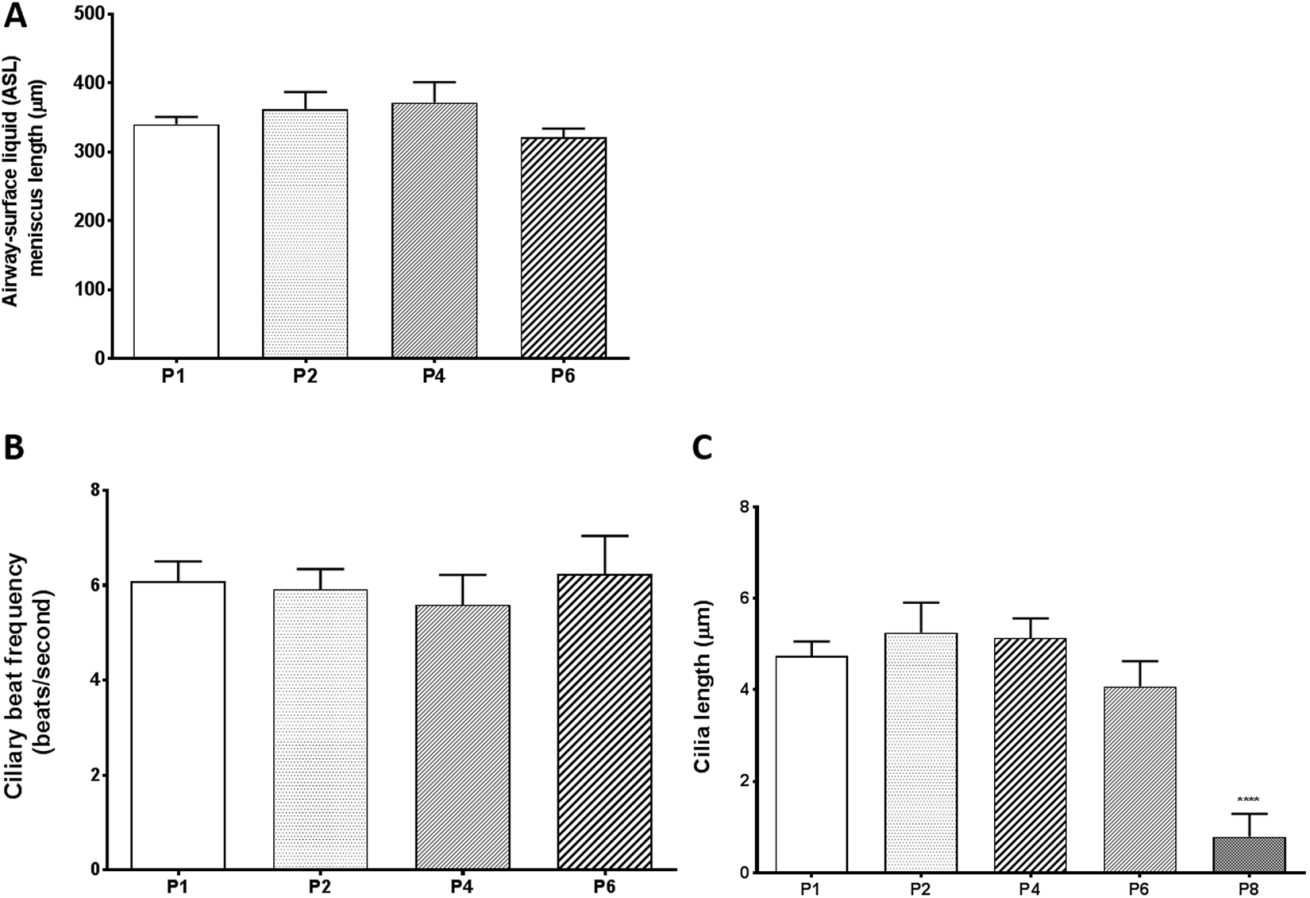
Effect of passage number on phenotypic characteristics including ASL, CBF, and cilia length. NHBE 3D culture characteristics were measured upon reaching 4 weeks at ALI. (**A**) ASL of 3D NHBE passaged cultures. (**B**) CBF of NHBE passaged 3D cultures, expressed in beats/second. (**C**) Cilia length (µm) of NHBE passaged 3D cultures. Data collated from 3 different donors, n = 3 for each donor. Significance compared to Passage 1, ****p < 0.0001.

As a part of mucociliary clearance, ASL and CBF were recorded for 3D epithelial cultures grown from passaged cells. Overall, no significant changes in ASL (as represented by meniscus length) was observed in epithelial layers from NHBE cells up to passage six (Fig. 8A). Similarly, no significant decrease in CBF was observed up to passage six (Fig. 8B). Finally, no difference in cilia length was observed up to passage six. However, cilia length was significantly decreased at passage eight (Fig. 8C).

## Discussion

For the first time, this study demonstrates that normal epithelial phenotypic characteristics are maintained in passaged primary NHBE cells, including crucial CFTR ion channel function. The ability to expand primary NHBE cells and form 3D epithelia for *in vitro* models is an important factor that can be implemented to a wide range of airway epithelial studies. In addition, we were able to compare different conditions to expand primary human airway cells and found that PneumaCult-Ex Plus (M3) medium did not require presence of feeder cells or ROCK inhibitor. Currently, in *in vitro* studies, passage one primary NHBE cells pose a limitation on resources and study design. Beyond passage one, primary cells begin to lose differentiation capabilities (ciliated and goblet cells) and fail to form 3D cultures. However, the ability to expand primary cells that can still form fully differentiated epithelium comparable to passage one cultures enables supplementary research that can be conducted for disease modeling, drug discovery and development of robust airway epithelial models from patients where samples are difficult or limited, such as infants/neonates and diseased patients^26^. The use of primary human cells as an *in vitro/*organotypic model increases the predictability of cellular responses to pollutants and onset of disease (e.g. COPD, CF), as well as drug efficacy and toxicity before moving into clinical trials^7^.

By comparing three different expansion media conditions, we identified that PneumaCult-Ex Plus (M3 medium) gave the best conditions for expanding primary human bronchial epithelial cells which retained differentiation properties when grown at ALI. Primary NHBE cells could be passaged up to 7–12 times (within 30 days) with M1 + ROCK or M3 before significant declines in growth was observed. Fully differentiated epithelial layers were consistently grown from cells passaged up to four times from 3 different donors (4 weeks ALI in PneumaCult-ALI differentiation medium). Crucial phenotypic and functional characteristics were maintained in the epithelial layers derived from passaged cells, including TEER > 400 Ohms.cm^2^, presence of ciliated and goblet cells, as well as CFTR function, ASL, and CBF similar to passage one. These endpoints are crucial factors in experimental design and investigating variability, particularly when studying the airway epithelium *in vitro*.

The added benefits of using Rho kinase inhibitor (ROCK) and mitotically inactivated 3T3-J2 feeder cells have been observed in another study when expanding sufficient numbers of primary tracheal airway cells for airway tissue engineering^23^. They also observed and characterized the decline of cell growth and differentiation capability when passaging cells in BEGM medium only, indicating that the self-renewal capabilities begins to be lost after 1–2 passages. Another study similarly used ROCK and conditioned BEGM media from irradiated inactivated NIH-3T3 feeder cells to conditionally regulate pediatric cells, passaging cells up to 5–6 times over 30 days and maintaining robust immune responses in differentiated passage five cells^26^. In this study, M3 medium (without addition of ROCK or 3T3 feeder cells) could similarly be used to expand primary human NHBE cells up to 7–9 passages in less than 30 days, further and faster than other published findings thus far.

In this study, 3D cultures were only achieved within 4 weeks at ALI from primary NHBE cells passaged in M3 medium (not M1 + ROCK medium). The M1 + ROCK medium grown NHBE cells may not have grown successfully at ALI due to the media change to PneumaCult-ALI (manufactured by a different company than BEGM). B-ALI medium (LONZA) was not commercially available due to a heavy backlog; hence, NHBE cells were only airlifted in PneumaCult-ALI medium. Unfortunately as of today, B-ALI is still not available.

Differences in the ability to grow 3D cultures were also observed between different donors; however, 3D cultures were optimally grown from cells up to passage six. Key differences between donors were observed in 3D cultures derived from NHBE cells beyond passage six, for example, donor A maintained the ability to form a pseudostratified epithelium with differentiated goblet and ciliated cells, optimal TEER and functioning CFTR ion channels (Figs 5A,B and 7B). On the other hand, donors B and C failed to form a pseudostratified epithelium with passage eight NHBE cells, indicating the variability between donors. It is therefore crucial to understand that different donors may retain variation in differentiation capabilities. However, importantly, epithelium integrity (TEER), ASL, CBF and cilia length did not significantly differ between passages one and six in 3D cultures among all three donors. CFTR ion channel function also remained stable. It is also theorized that various harvesting protocols and cryogenic storage of passage zero harvested donor cells will play an important factor in the success of using cells for expansion. These factors, such as cryogenic preservation media and cell harvesting protocols were not investigated in this study as the original focus was on characterizing the differentiated cells.

Furthermore, we have been able to observe a gradual disintegration of the airway epithelium grown from passaged cells beyond passage six, particularly with columnar epithelial structure (Fig. 5A), cilia length (Fig. 8C) and epithelial integrity (Fig. 5B). This highlights the limitation of using higher passage number primary cells in *in vitro* models compared to cell-lines which can be technically passaged indefinitely^28^. However, primary cells are optimal models to represent the native and original cells with their ability to differentiate into ciliated cells, goblet cells and club cells. Similar to what has been described in ‘normal’ lungs^1,2^, 3D cultures in this study demonstrated an estimated 70–80% ciliated cells and 15–26% goblet cells (MUC5B and MUC5AC) (Fig. S6).

Although CFTR ion channels did not appear to significantly change in our 3D cultures from passaged cells, a significant decline in ENaC was observed beyond passage one. ENaC has been attributed to the pathology of cystic fibrosis (CF), and there has been suggestions that ENaC and CFTR are “functionally interrelated” due to the close proximities of these epithelial ion channels^29^. Impaired function of ENaC and CFTR can result in dehydration of the airway surface fluid, often seen in CF patients. We, however, have not detected a decline in airway surface fluid (Fig. 8A) but it could be due to the decrease in ENaC function as sodium hyper-absorption through ENaC has been reported in CF. In addition, ENaC has also been shown to be expressed on the cilia of the airway epithelium^30^. However, we did not detect any change in CBF or ASL. Future studies will be required to determine whether the decline in ENaC function is due to decreased expression of ENaC subunits or reduced activation of ENaC channels from the surface of ciliated cells and/or in cilia since this channel is activated after cleavage of the α and γ subunits by proteases^31^. Therefore, depending on the experimental design it is important to determine whether passaged primary cells will affect certain aspects of the 3D cultures.

Future investigations into the use of passaged primary human airway cells for airway-on-a-chip models as well as co-culture conditions may also be of interest^32^. In addition, the combination of primary passaged cells and automated cultivation systems could further enhance the large-scale expansion of primary airway cells for high-throughput screenings^33^.

In conclusion, the phenotypic endpoints evaluated in this study are critical for the development of *in vitro* lung models. It is necessary to optimize and choose appropriate culture characteristics for consistent results. This study has demonstrated primary NHBE cells can be passaged up to 6–8 times, and that normal epithelial phenotypic characteristics are maintained in passaged primary NHBE cells up to passage four, including crucial CFTR ion channel function which is important for airway mucociliary clearance.

## Methods

### Primary Human Airway Epithelial Cells

Primary normal human bronchial epithelial (NHBE) cells were either sourced from LONZA (NHBE CC-2540s; Walkersville, MD) from a non-smoking patient or were gifted from Nationwide Children’s Hospital Cell Core, Columbus OH (3 donors) without identifiers (exempt status from the Institutional Review Board).

### Cell Culture

#### Cell expansion

Three different initial proliferation culture media were used to revive NHBE (LONZA) cells from cryopreservation, seeded as passage one (P1) into T25 cell culture tissue flasks: BEGM growth media (LONZA, Walkersville MD), PneumaCult-Ex (PnC-Ex) media (StemCell, Tukwila WA) or PneumaCult-Ex Plus (PnC-Ex-PLUS) media (StemCell, Tukwila WA). Cells were seeded at ~100,000 cells/T25 flask, and incubated at 37°C, 5% CO_2_. Once cells reached 70–80% confluency, they were dissociated using TryplE Express dissociation media (ThermoFisher, MA USA). From passage two (P2), different growth conditions were tested, including the use of a Rho kinase inhibitor (Y-27632; R&D Systems, Minneapolis MN) and/or feeder cells (mouse 3T3 fibroblast cells treated with mitomycyin C) (Fig. S1).

#### Cells cultured at Air-Liquid Interface (ALI)

Once cells had reached 70–80% confluency, they were dissociated and seeded on 6.5 mm Transwells (Corning; Fisher Scientific) coated with 0.3 mg/mL Collagen type IV from human placenta (Sigma-Aldrich, St. Louis MO) (Fig. S1). NHBE cells grown in BEGM + ROCK, PnC-Ex or PnC-Ex-PLUS media were seeded at 50,000 cells/well. Respective growth media was used to feed cells until 4–5 days or 100% confluency was reached. Upon reaching confluency, the apical media was removed and the basal media replaced with PneumaCult-ALI medium for all cell conditions. Medium was changed every second day and apical surfaces washed with HBSS (Ca^2+^, Mg^2+^) twice per week. All cells were grown until 4 weeks airlifted (37°C, 5% CO_2_) before various physiological and characterization tests were performed (Fig. S1). Epithelial layers were grown from passaged cells until they failed to expand any further.

### Hematoxylin & Eosin (H&E) and periodic acid-Schiff /alcian Blue (PAS/AB) Staining

Upon 4 weeks airlifted, NHBE cells were fixed in 4% paraformaldehyde, embedded and sectioned for H&E staining. Similarly, NHBE 3D cultures were also fixed in Carnoy’s fixative solution overnight, placed in 4% paraformaldehyde, embedded and sectioned for PAS/AB staining.

### Immunofluorescence Staining

NHBE cells fixed in 4% paraformaldehyde were embedded in paraffin and sectioned for immunofluorescence staining. Certain epithelial features were tested including α-tubulin (B-5-1-2#322588 Alexa Fluor-488 conjugated, Invitrogen, 1:500), MUC5AC (clone 45M1 #M5293 Sigma Aldrich, 1:100), MUC5B (#HPA008246, Sigma Aldrich, 1:100), and ZO-1 (1A12, #339194 Alexa Fluor-594 conjugated, Invitrogen, 1:100). Respective secondary antibodies were used for the primary antibodies not already conjugated to a fluorophore. Briefly, paraffin embedded epithelial sections were de-parafinized with xylene and ethanol washes, before permeabilizing and subjected to antigen retrieval. Cells were blocked with 1% BSA/10% Normal Goat serum in PBS + 0.05% Tween-20 (PBST). Primary antibodies (including those already conjugated to a fluorophore) were diluted in PBST and incubated with the cells at room temperature in a humidified chamber overnight. Cells were washed with PBST and secondary antibody was incubated at room temperature for 1.5 hours in the dark in a humidified chamber. Cells were washed again with PBST before staining with Prolong Gold anti-fade with 4′, 6-diamidino-2-phenylindole (DAPI) (Invitrogen, Waitham, MA) and imaged with a fluorescent microscope.

### Trans-Epithelial Electrical Resistance (TEER)

TEER of primary NHBE 3D cultures were measured using EVOM resistance meter at 4 weeks post-lifting to ALI.

### Airway surface liquid (ASL), ciliary beat frequency (CBF), and cilia length

Airway surface liquid (ASL) meniscus length was measured using ImageJ analysis of imaged apical surfaces (x10 objective) following Myerburg’s protocol^34^. Briefly, the shadow of the ASL meniscus can be visualized, and length measured as a reflection of liquid volume. Ciliary beat frequency (CBF) was recorded by imaging the apical surface (X60 objective) with a camera with slow-motion capture 720pixels at 240fps (frames-per second). Analysis was performed using ImageJ. Cilia length was measured off H&E sections (n = 3 sections per donor), averaging the length of 20 cilia per section.

### Short-circuit Current Measurements

To assess ion channel function, specifically Epithelial Sodium Channel (ENaC) and Cystic Fibrosis Transmembrane Conductance Regulator (CFTR) channel function, NHBE cells were mounted into Ussing chambers (system VCC MC6 from Physiologic Instruments, Inc. San Diego, CA) with U2500 Self-contained Ussing chambers (Warner Instruments, Hamden CT) as previously described by our group^35^. Ringer’s solution was placed on the apical and basal side of the transwell. Once current was stabilized, a low chloride solution was exchanged on the apical side with Ringer’s maintained on the basal side. Following this, 100 μM amiloride was added apically to inhibit ENaC function. Forskolin (10 μM; Abcam, Cambridge MA) was later added apically to activate CFTR channels via activation of adenylyl cyclase and then inhibited with CFTR inh-172 (10 μM) (Sigma Aldrich, St. Louis MO).

### Data Analysis

All data analysis and statistical calculations were performed using GraphPad Prism^®^ v7.04 (GraphPad Software, Inc.) and are expressed as mean ± SEM. Statistical significance was determined using Two-tailed t-test compared to control with 95% confidence interval.

## Electronic supplementary material

Supplementary Data

## Data Availability

All data generated or analyzed during this study are included in this published article (and its Supplementary Information files).
