# Supplementary material for: Optimization of Normal Human Bronchial Epithelial (NHBE) Cell 3D Cultures for in vitro Lung Model Studies
Source: Sci Rep. 2019 Jan 24;9:500. doi: 10.1038/s41598-018-36735-z (PMC6346027; doi:10.1038/s41598-018-36735-z)
Supplement: Supplementary file 1 — Supplementary Data [file 41598_2018_36735_MOESM1_ESM.pdf]

Optimization of Normal Human Bronchial Epithelial (NHBE) Cell 3D Cultures for *in vitro* Lung Model Studies

Rachael E. Rayner[1], Patrudu Makena[2], Gaddamanugu L. Prasad[2] & Estelle Cormet-Boyaka[1, *]

[1] The Ohio State University, Department of Veterinary Biosciences, Columbus, OH 43210
[2] RAI Services Company, Winston-Salem, NC 27101

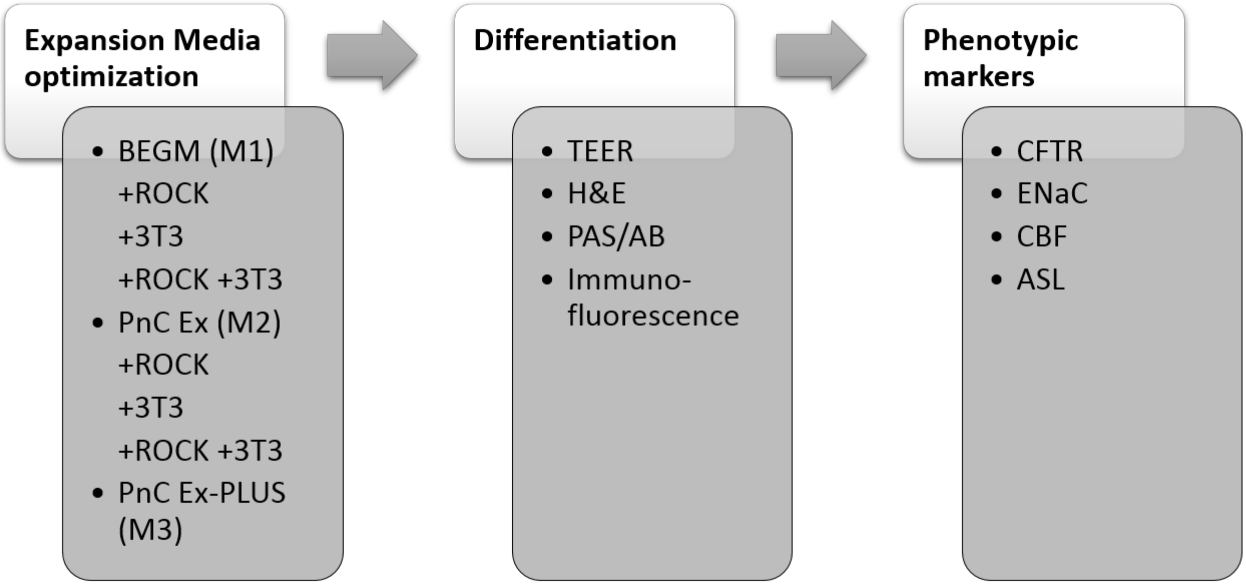

Supplementary figure 1. Stages of experimental design, from expansion media optimization through to characterizing differentiated passaged primary NHBE cells. Three expansion mediums, Bronchial Epithelial Growth Medium (BEGM; M1), PneumaCult-Ex (PnC Ex; M2) and PneumaCult-Ex Plus (PnC Ex-PLUS; M3) were grown with/without Rho kinase inhibitor (ROCK) and/or 3T3 Swiss fibroblast feeder cells (3T3). Differentiation of NHBE cells in ALI was performed. Verification of differentiated epithelial layers was determined using Trans-epithelial electrical resistance (TEER), hematoxylin and eosin (H&E) staining, Periodic acid-Schiff/Alcian blue (PAS/AB) assay and immunofluorescence. Other phenotypic markers including functions of Cystic fibrosis transmembrane conductance regulator (CFTR), Sodium Epithelial Channel (ENaC), ciliary beat frequency (CBF) and airway surface liquid (ASL) were also characterized.

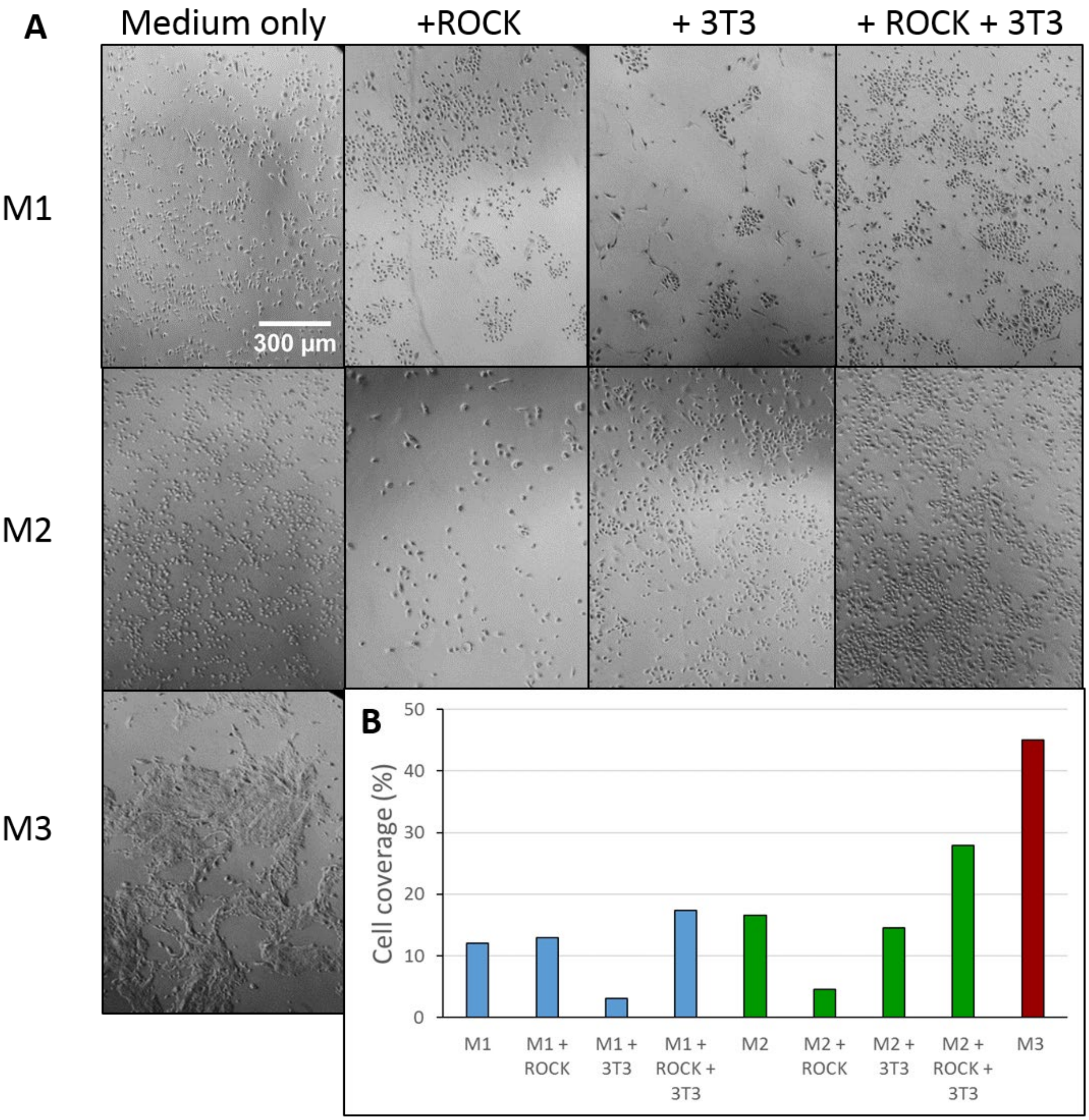

Supplementary figure 2. Morphology of expanded primary NHBE cells in various expansion conditions. Expansion of NHBE cells (passage two, 4 days growth) in growth medium only, or with Rho kinase inhibitor (ROCK) and/or 3T3 fibroblasts. Same number of cells were plated for each condition. A) Microscopy images of cell growth after 4 days showing scattering or clustering of cell colonies. B) Percentage coverage of cells after 4 days growth in expansion conditions. M1 – BEGM; M2 – PneumaCult-Ex; M3 – PneumaCult-Ex Plus. Objective X200.

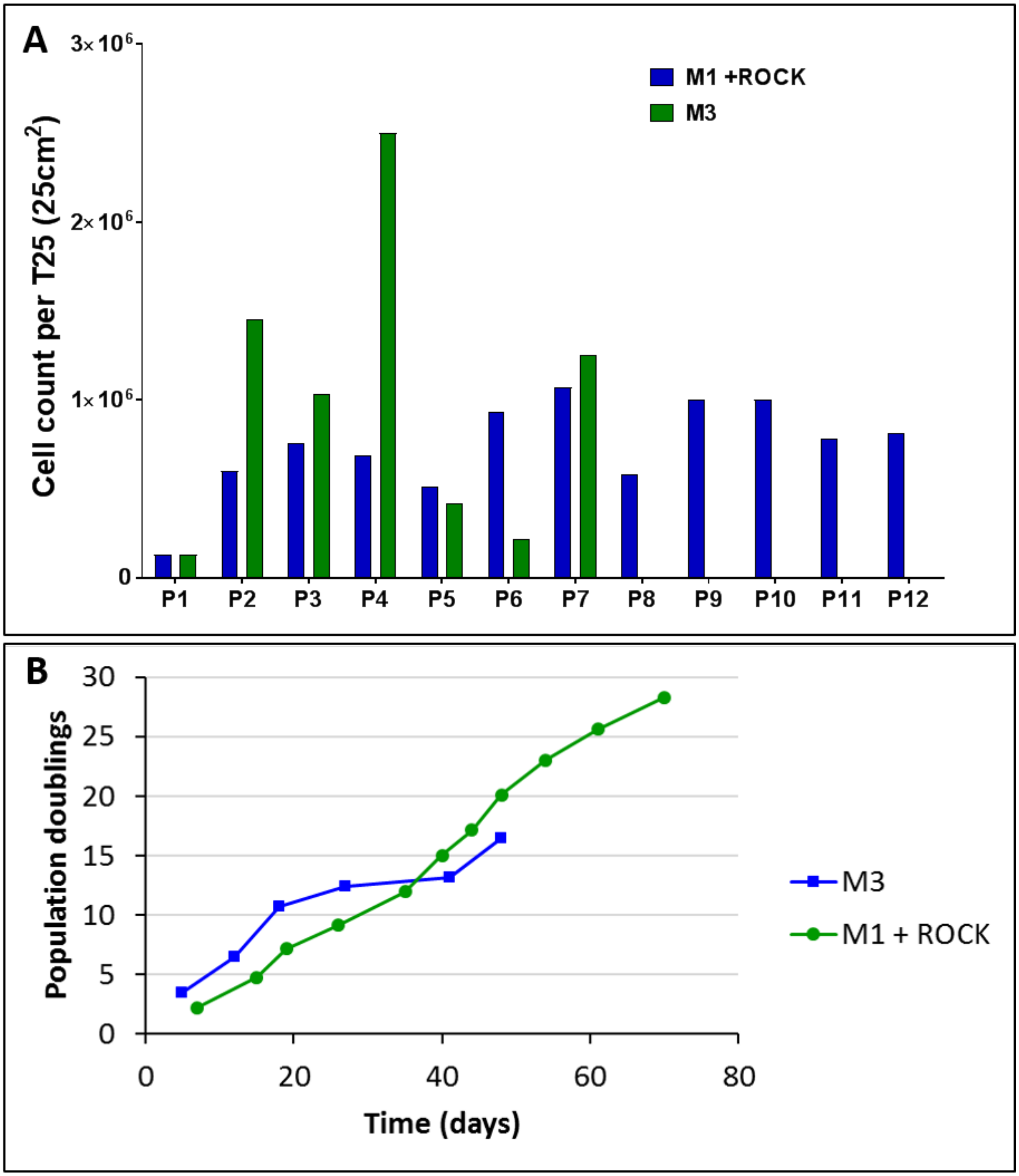

Supplementary figure 3. Expansion of primary NHBE cells in two culture conditions. A) Time for primary NHBE cells to reach ~75% confluency in M1+ROCK or M3 media. B) Population doubling of primary NHBE cells in M1+ROCK or M3 media. N = 2.

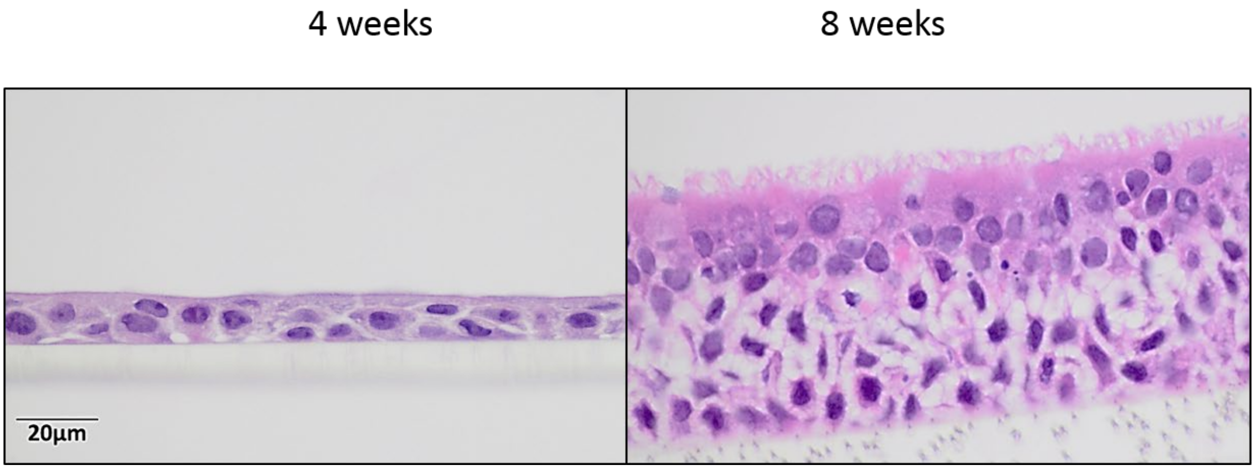

Supplementary figure 4. Differentiation of primary NHBE cells expanded in M2 medium. 3D epithelium of NHBE cells grown for 4 and 8 weeks in ALI, from cells expanded in M2 medium to passage 2.

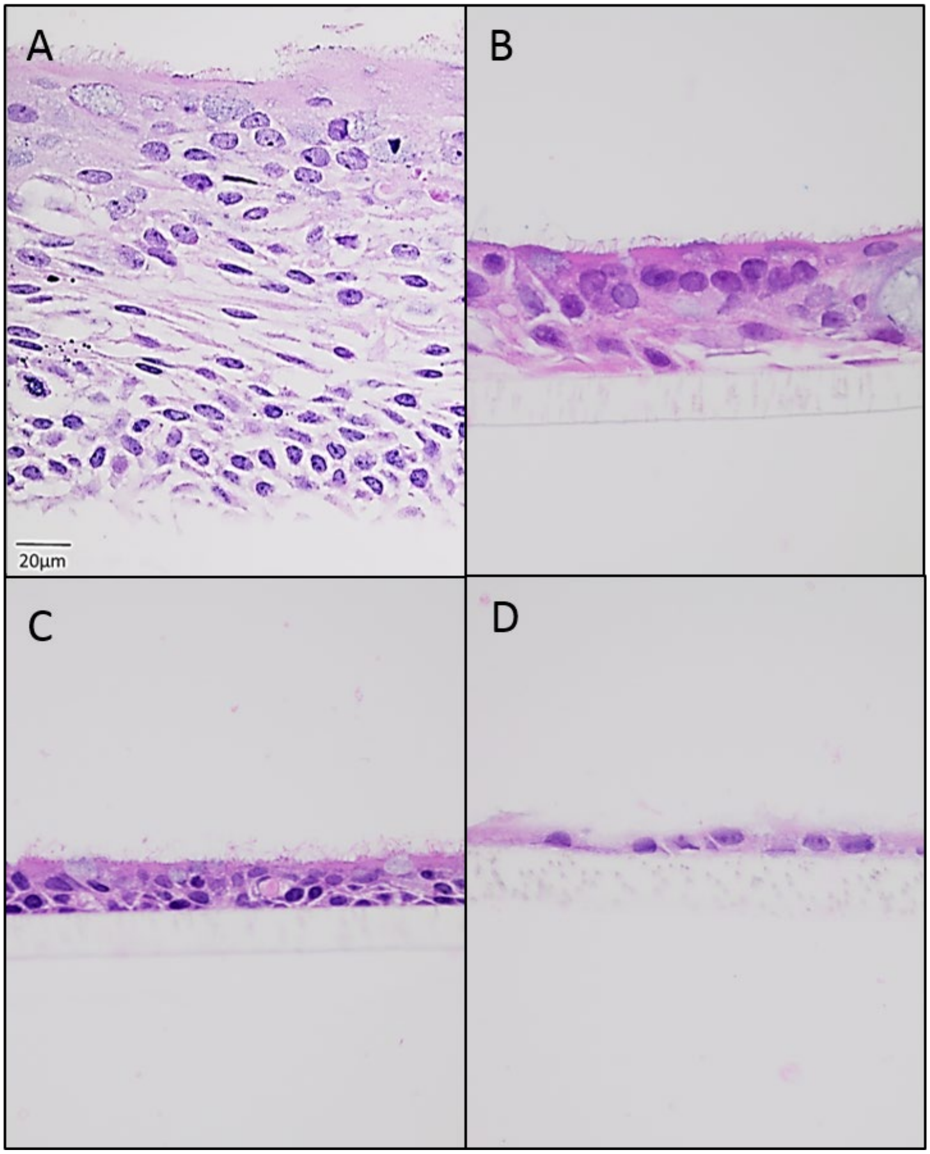

Supplementary figure 5. Differentiation of primary NHBE cells. NHBE (passage 2) were seeded in M3 medium at various seeding densities prior to differentiating in ALI medium. A) 100,000cells/well, B) 50,000cells/well, C) 25,000cells/well, D) 20,000cells/well. H&E staining of epithelial layers was performed post 4 weeks ALI. Objective X400.

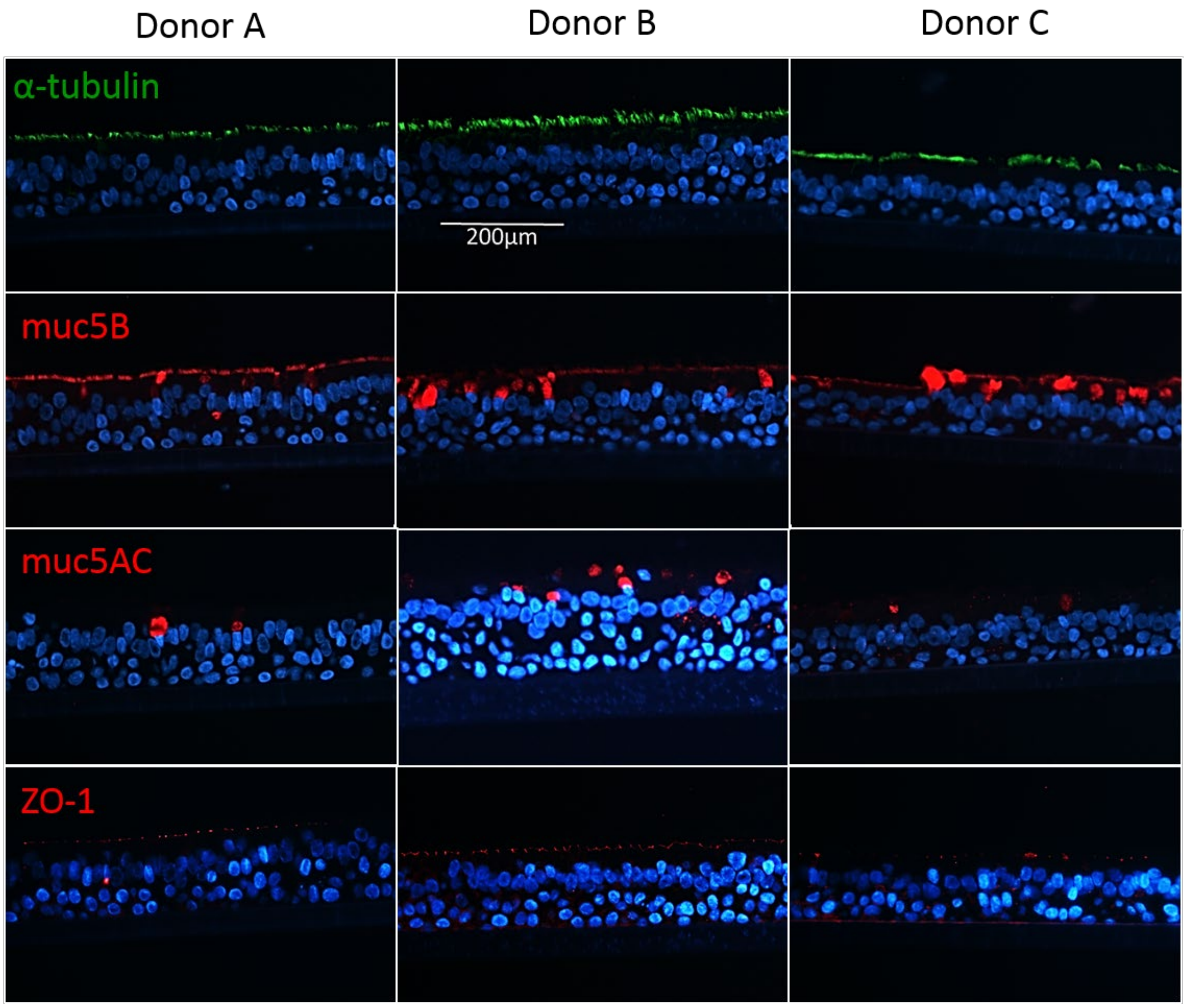

Supplementary figure 6. Differentiation of primary NHBE 3D cultures from passage 1 cells grown in M3 medium. Immunofluorescent staining of passaged NHBE cells from three donors shows presence of ciliated cells (α-tubulin), goblet cells (muc5B and muc5AC), and tight junctions (ZO-1) (4 weeks at ALI passage 1; X400 objective).
